# Gut Microbial Dysbiosis and Plasma Metabolic Profile in Individuals With Vitiligo

**DOI:** 10.3389/fmicb.2020.592248

**Published:** 2020-12-14

**Authors:** Qingrong Ni, Zhubiao Ye, Yinghan Wang, Jianru Chen, Weigang Zhang, Cuiling Ma, Kai Li, Yu Liu, Ling Liu, Zheyi Han, Tianwen Gao, Zhe Jian, Shuli Li, Chunying Li

**Affiliations:** ^1^Department of Dermatology, Xijing Hospital, Fourth Military Medical University, Xi’an, China; ^2^Department of Gastroenterology, Xijing Hospital, Fourth Military Medical University, Xi’an, China; National Clinical Research Center for Digestive Diseases, Xi’an, China

**Keywords:** vitiligo, gut microbiome, 16S rRNA sequence, serum metabolomic, gut-skin axis

## Abstract

Autoimmune diseases are increasingly linked to aberrant gut microbiome and relevant metabolites. However, the association between vitiligo and the gut microbiome remains to be elucidated. Thus, we conducted a case-control study through 16S rRNA sequencing and serum untargeted-metabolomic profiling based on 30 vitiligo patients and 30 matched healthy controls. In vitiligo patients, the microbial composition was distinct from that of healthy controls according to the analysis on α- and β-diversity (*P* < 0.05), with a characteristic decreased *Bacteroidetes: Firmicutes* ratio. Meanwhile, the levels of 23 serum metabolites (including taurochenodeoxycholate and L-NG-monomethyl-arginine) in the vitiligo patients were different from those in the healthy individuals and showed significant correlations with some microbial markers. We found that *Corynebacterium 1*, *Ruminococcus 2*, *Jeotgalibaca* and *Psychrobacter* were correlated significantly with disease duration and serum IL-1β level in vitiligo patients. And *Psychrobacter* was identified as the most predictive features for vitiligo by machine learning analysis (“importance” = 0.0236). Finally, combining multi-omics data and joint prediction models with accuracies up to 0.929 were established with dominant contribution of *Corynebacterium 1* and *Psychrobacter*. Our findings replenished the previously unknown relationship between gut dysbiosis and vitiligo circulating metabolome and enrolled the gut-skin axis into the understanding of vitiligo pathogenesis.

## Introduction

Vitiligo is an autoimmune skin disease affecting 0.5 to 1% population worldwide. The disease is characterized by the loss of pigment resulting from the massive melanocytes destruction (Dell’Anna et al., 2007; Ezzedine et al., 2015; Bae et al., 2018).

Currently, adaptive immunity activated by melanocytes-specific antigens is the major focus of the researches on vitiligo pathogenesis (Li et al., 2017). The autoreactive CD8^+^ T cells is appreciated as the dominant player in melanocyte destruction. And the activation of CD8 is also modulated by various factors like the enhanced inflammatory microenvironment precipitated by excessive proinflammatory factors. IL-1β is such a cytokine. CD4^+^ T cells like Th1 and Th17 cells are documented to assist the aberrant response of CD8^+^ T cell in vitiligo (van den Boorn et al., 2009; Kotobuki et al., 2012; Czarnowicki et al., 2019). Besides, declined frequency and aberrant function of regulatory T cells (Tregs) give rise to insufficient restraint on CD8^+^ T cells (Zhou et al., 2012). Recent studies have demonstrated that serum level of soluble interleukin (IL)-2 receptor a (CD25) reflects T-cell activation in vitiligo (Speeckaert et al., 2016), whereas IL-17-induced secretion of IL-1β from keratinocytes links with autophagic melanocytes apoptosis (Zhou et al., 2018). These factors might synergistically induce melanocyte paucity and perpetuate vitiligo, though the exact trigger of autoimmune disorder in vitiligo is still ill-defined. And these important serum molecules are also commonly used as biomarkers for vitiligo activity (Speeckaert et al., 2016, 2017; Bhardwaj et al., 2017).

Recent insights have defined the critical role of the gut microbiome in keeping immune homeostasis and in the development of autoimmune diseases (Cohen et al., 2019; Dominguez-Bello et al., 2019). Although the interplay between the intestinal microbiome and the autoimmune state has never been investigated in vitiligo, a putative connection between vitiligo and intestinal microbiome essentially exists. It has been noted that vitiligo patients have a high rate of comorbidity with inflammatory bowel disease (IBD) (Hadi et al., 2019), another autoimmune disease associated with aberrant intestinal microbiome (Gevers et al., 2014; Lloyd-Price et al., 2019). Furthermore, the proposed concept of the gut-skin axis provides a plausible correlation between gut microbiome and dermatoses, which has been reiterated in two autoimmune skin diseases including psoriasis (Hidalgo-Cantabrana et al., 2019) and atopic dermatitis (Mahdavinia et al., 2019). Meanwhile, Dellacecca et al. explored the changes in pigmentation of vitiligo mice after the treatment of oral antibiotics (Dellacecca et al., 2020). They found that ampicillin treatment correlated with accelerated depigmentation, reduced bacteria in fecal pellets and changed distribution of T cells in tissues and blood, suggesting the association between gut dysbiosis and ampicillin-induced depigmentation. Meanwhile, the important clue is that IL-1β, which is closely related to vitiligo activity, not only participates in the modulation of immune programs (Warnatsch et al., 2017; Yao Y. et al., 2017), but also enhances local antimicrobial peptides to potentiate microbiome remodeling (Yao X. et al., 2017).

Intestinal metabolite profiles are derived from microbe-sourced compounds that dictate microbial-host interactions (Donia and Fischbach, 2015; Dodd et al., 2017; Liu et al., 2017). Small molecules produced by gut microbiome serve as critical mediators that prime the maturation and postnatal adaption of the host immune system (Fulde and Hornef, 2014) and even trigger autoimmune diseases (Cianci et al., 2018; Haase et al., 2018). Based on these previous findings, we hypothesized that the gut-skin axis could be involved in the pathogenesis of vitiligo via microbial metabolites. To testify this, 16S rRNA sequencing and serum metabolome analysis through non-targeted liquid chromatography-mass spectrometry (LC-MS) were performed in fecal samples from a cohort of 60 individuals. The interaction of vitiligo-related gut microbiome and serum metabolome was investigated and their role in the immune pathogenesis of vitiligo was further analyzed in the present study.

## Results

The study cohort encompassed 30 patients diagnosed with advanced non-segmental vitiligo (12 men, 18 women, mean age 37.2 ± 12.7 years old) as well as 30 age-, sex-, Body Mass Index- (BMI-) and dietary habit-matched healthy controls (12 men, 18 women, mean age 35.2 ± 12.5 years old) from 2018 to 2019. The severity of the disease is often evaluated by calculating the white spot area [like Vitiligo Extent Score (VES) (van Geel et al., 2018) and Vitiligo Area Scoring Index (VASI) (Komen et al., 2015)] clinically. All patients underwent VES evaluation. And the average VES of the patients was 6.2%. Detailed clinical characteristics of the study population are presented in Supplementary Table 1.

### Vitiligo Patients Exhibit Gut Microbiome Dysbiosis

**FIGURE 1:**
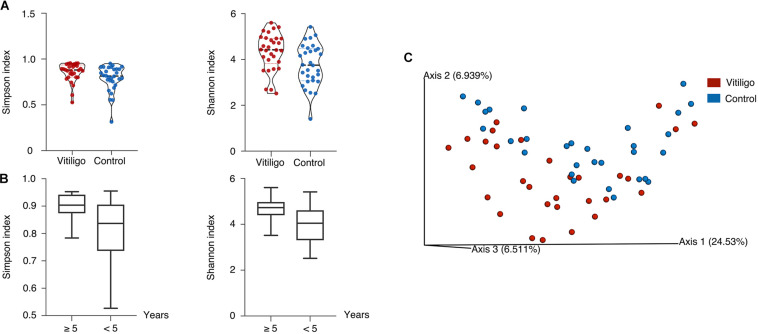
Distinct gut microbial diversity in vitiligo. **(A)** Compared with the controls, gut microbial α-diversity, as estimated by the Shannon index and Simpson index, was significantly increased in patients with vitiligo (*P* = 0.014, 0.027, respectively, Kruskal-Wallis pairwise test). **(B)** The Shannon and Simpson indexes in ≥5 years showed significantly higher than <5 years group (*P* = 0.020, 0.038, respectively, Kruskal-Wallis pairwise test). **(C)** Microbial clustering was shown based on unweighted PCoA metrics using 3D plots, indicating a symmetrical distribution of gut microbial community among all the samples. Significant dissimilarity distances were found (*P* = 0.015, PERMANOVA). Abbreviation: PCoA, Principal Coordinate Analysis; PERMANOVA, Permutational Multivariate Analysis of Variance.

To characterize the overall gut microbiota composition of vitiligo patients, we sequenced the V4 region of the bacterial 16S rRNA gene in fecal samples collected from our cohort. Reads with ≥99% nucleotide sequence identification were grouped into Amplicon Sequence Variants (ASVs). We measured α- and β-diversity to delineate discrepancies between vitiligo patients and healthy controls on gut microbiome composition. The vitiligo microbiome samples had higher Shannon and Simpson indexes than control ones (Kruskal-Wallis pairwise test, *P* < 0.05, Figure 1A, Supplementary Figure 1 and Supplementary Table 2), indicating increased gut microbial diversity in diseased individuals. The patients are segregated into two groups by their disease duration (5 years below and 5 years more, respectively). We found that the Shannon (Kruskal-Wallis pairwise test, *P* = 0.020) and Simpson (Kruskal-Wallis pairwise test, *P* = 0.038) indexes between the two groups showed significant difference, and that the α-diversity for patients with over 5-year disease course was significantly higher (Figure 1B). In Principal Coordinate Analysis (PCoA) of the unweighted UniFrac 3D plot chart, our samples were divided into two groups (Figure 1C, PEMANOVA test, *P* < 0.05, also see in Supplementary Table 2), with PCA1 contributing the most and reaching 24.53%. To summarize, gut dysbiosis were detected in vitiligo patients compared with healthy controls.

### Several Bacterial Taxonomies Significantly Contribute to Vitiligo Gut Microbiome Dysbiosis

**FIGURE 2:**
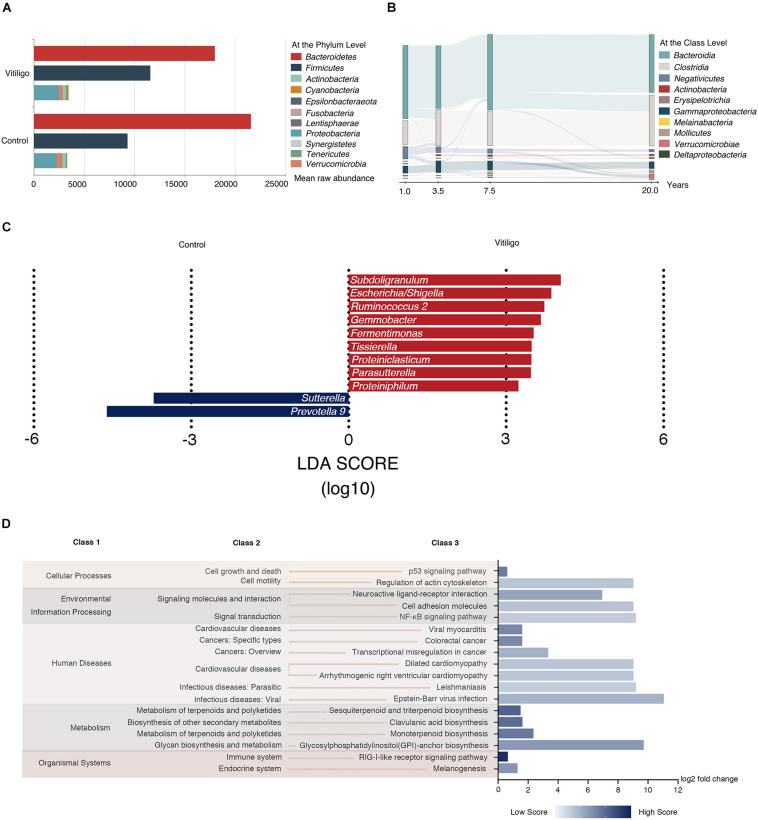
Taxonomy composition and Tax4fun predictive function results of gut microbiome in vitiligo. **(A)** Mean raw abundance of fecal microbiome at the phylum level between vitiligo patients and healthy controls, for taxa with >1% mean raw abundance across all samples. Taxa with open bars show differential *Bacteroidetes* to *Firmicutes* raw abundance ratio between cohorts. **(B)** Patterns of gut microbiome mean raw abundance at the class level dynamics with the duration of disease were tracked using Sankey plots in vitiligo patients. **(C)** Microbial markers at the genus level in vitiligo by using LEfSe analyses. LEfSe analyses revealed that the relative abundances of 11 genera were significantly different between vitiligo patients and the control group. The abscissa represents LDA score (log10). **(D)** Tax4fun is used to infer the functional content of the gut microbiome based on 16S data. Fold change of the abundance of KEGG pathways between vitiligo patients and healthy controls assessed by the Wilcoxon signed-rank test with FDR < 0.05. The relative abundance score of each differential pathway enrichment is reflected by the color depth. The abscissa represents log2 fold change value of relative abundance score. Abbreviation: LDA, Linear Discriminant Analysis; KEGG, Kyoto Encyclopedia of Genes and Genomes; FDR, False Discovery Rate.

The taxonomic assignment of the ASVs predicted for all the 60 samples revealed the composition of their bacterial population at the genus level. Back to the phylum level, vitiligo patients showed a lower average representation of *Bacteroidetes* (54.4 vs 63.1%, *P* < 0.05) and higher rate of *Firmicutes* (35.0 vs 27.2%, *P* < 0.05) according to Analysis of Variance (ANOVA), as well as lower *Bacteroidetes*: *Firmicutes* ratio (1.6: 1) versus healthy controls (2.3: 1). Besides, the mean raw abundance of *Negativicutes* was higher and the mean raw abundance of *Bacteroidia* was lower in patients with vitiligo at the class level. No significant discrepancy was observed in the rest data (Figure 2A and Supplementary Table 3).

We then separated the patients into 4 subsets according to disease duration (<2, 2–4, 5–9, and 10–30 years) and analyzed the composition of their gut microbiome species at the class level. We found that the species alterations were correlated with disease duration. Notably, the mean raw abundance of *Negativicutes* showed a slow decline as the disease course prolonged (Figure 2B), while the raw abundance of the remaining classes was not considerably changed. Moreover, we observed dynamic shifts according to VES in the mean raw abundance of some class such as *Negativicutes*, *Bacteroidia*, and *Clostridia* (Supplementary Figure 2). LEfSe analysis revealed that *Subdoligranulum*, *Escherichia/Shigella*, *Ruminococcus 2*, and *Gemmobacter* were dominant biomarkers to distinguish vitiligo patients from healthy controls at the genus level (Figure 2C and Supplementary Table 4). To provide further insights into the microbial community structure in vitiligo patients, Meanwhile, we set up a co-occurrence network across the top microbial markers (Linear Discriminant Analysis (LDA) score >2.20) via LEfSe and observed 155 significant associations in the network (*P* < 0.05, Supplementary Figure 3).

Subsequently, potential functions of the gut microbiome in vitiligo were predicted via Tax4Fun analysis (Asshauer et al., 2015) with a custom database. We identified at least 265 Kyoto Encyclopedia of Genes and Genomes (KEGG) orthologs (KOs) appearing in each sample, among which 66 KOs differed in abundance between vitiligo patients and healthy subjects (*P* < 0.05, Supplementary Table 5). Of note, KEGG pathways marked with “Epstein-Barr virus infection” involved in “infectious diseases: viral” were highly enriched in the microbiome of vitiligo individuals. Moreover, “regulation of actin cytoskeleton” involved in “cell motility” and “NF-κB signaling pathway” in “signal transduction” were significantly enriched in vitiligo patients compared with controls, which merits follow-up research for verification (Figure 2D).

### *Psychrobacter* Is the Most Predictive Features in Microbial Machine Learning Model of Vitiligo

**FIGURE 3:**
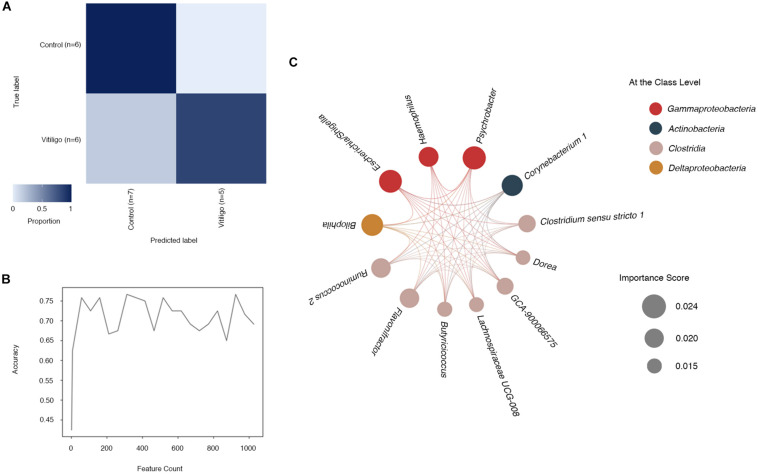
Identification of microbial genus-based markers of vitiligo by machine learning method. **(A)** “Confusion matrix” heat map shows classifier accuracy results at the ASV level. Numbers marked in the figure represent the size of the test set, which is randomly split by learning methods. This “Confusion matrix” shown in figure was analyzed from a test set of 24 samples (11 vitiligo patients and 13 healthy controls) and a learning set of 36 samples. **(B)** The relationship between the number of different features and the prediction accuracy is represented by a line graph. **(C)** Co-occurrence patterns of the most important feature selected from the output of supervised learning methods at the genus level are visualized using network diagrams. Microbial taxa are represented by nodes and complex intertwined lines denote positive co-occurrence relationships based on correlation. Each co-occurrence relationship had strong Spearman’s correlation coefficient (*P* < 0.05). Circle size represents the importance score of selected features and circle color represents the class to which each genus belongs. Abbreviation: ASVs, Amplicon Sequence Variants.

Regarding the observed particularity of gut microbiome in vitiligo, we explored the potential of microbial taxonomy markers to be diagnostic predictors of vitiligo. The machine learning technique was adopted and the microbial composition displayed a high prediction accuracy at the ASVs level (91.7%), which was slightly higher than that at the genus level (83.3%) as depicted in the confusion matrix (Figure 3A, Supplementary Figure 4 and Supplementary Table 6). Model accuracy approached 70% with modest fluctuations as the number of features increased (Figure 3B). Data presented herein indicated that gut microbiome could be employed to distinguish vitiligo from healthy populations.

To determine the most predictive features in our supervised learning model, we enrolled feature “importance” scores as a parameter (Pedregosa et al., 2011). Features at the genus level with reported higher “importance” scores (Supplementary Table 7) contributed more to the discrimination of vitiligo samples. The candidate genera included pertaining to *Psychrobacter* with the highest score of 0.0240, followed by *Escherichia/Shigella* (“importance” = 0.0236), *Bilophila* (“importance” = 0.0226) and *Corynebacterium 1* (“importance” = 0.0218), as shown in the model. Intriguingly, some of the most predictive genera in the screening also performed well in the LEfSe analysis, such as *Psychrobacter* (LDA = 2.955, *P* < 0.01), *Escherichia/Shigella* (LDA = 3.862, *P* < 0.01) and *Corynebacterium 1* (LDA = 2.896, *P* < 0.01, Supplementary Table 4). *Subdoligranulum*, the most potential vitiligo-associated genus as indicated by previous LEfSe analysis, however, failed to be identified by our machine learning model.

Then, we selected genera with relatively higher “importance” scores (“importance” >0.015) and analyzed their co-occurring network (Figure 3C). The numbers of significant associations among these 12 genera markers were in equilibrium, with 128 significant ones altogether.

### *Corynebacterium 1*, *Ruminococcus 2*, *Jeotgalibaca* and *Psychrobacter* Are Correlated With Disease Duration and Serum IL-1β Level of Vitiligo

**FIGURE 4:**
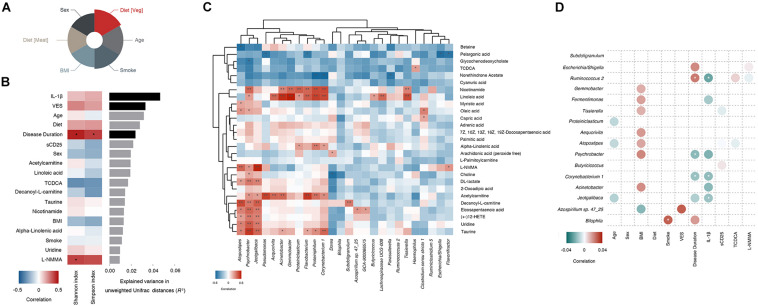
Clinical determinants correlate with the gut microbiome composition of vitiligo patients. **(A)** Contribution of each clinical covariate to gut microbial variation calculated by the regression model show in Nightingale’s Rose Diagram. **(B)** Related clinical determinants of intestinal microbial richness and microbiome composition in patients with vitiligo are shown. In the bar graph, the *x*-axis represents the interpretation variance for each phenotype consisting of the gut microbiome (unweighted Unifrac). Black bars indicate statistical significance (FDR < 0.1). The heat map indicates a significant positive correlation (red, *P < 0.05) or negative correlation (blue, *P < 0.05) between determinants and microbial richness (Shannon index) and bacterial gene richness (Simpson index). **(C)** Spearman’s rank correlation between gut microbiome markers (LDA score >2.00 and “importance” >0.015) and serum differential metabolites selected by VIP score (VIP > 1) with adjusted *P* < 0.05 are shown (*P < 0.05; **P < 0.01). The heat map indicates a significant positive correlation (red) or a negative correlation (blue). **(D)** Correlation between clinical determinants and significantly different genera (LDA score >2.50 and “importance” >0.021) is shown in plot heatmap. Each plot represents a significant coefficient (positive in red and negative in green, *adjusted *P*-value < 0.1 [after correction for the false discovery rate with Benjamini and Hochberg procedure)]. Abbreviation: LDA, Linear Discriminant Analysis; MaAsLin, Multivariate Association with Linear models; FDR, False Discovery Rate; IL-1β, Interleukin 1beta; VIP, Variable Importance for the Projection.

We calculated the relation between internal-external factors and the gut microbiome composition of vitiligo patients. The results revealed that multivariate response linear regression (Analysis using “Gneiss” method) explained 12.26% of the community variation, which was prototypical for data observed in the human gut microbiome. Diet (Veg) identified in the covariate analysis was the most remarkable explanatory factor, accounting for 1.78% of the variation (Figure 4A and Supplementary Table 8).

We identified 23 differential metabolites from 15 vitiligo patients and 15 controls in the cohort (Variable Importance for the Projection (VIP) >1 in Orthogonal Partial Least Squares Discriminant Analysis (OPLS-DA) model, *P* < 0.05 in One-way ANOVA test, Supplementary Table 9). Following that, we measured the correlation between characteristics in our cohort (Figure 4B, Supplementary Tables 10, 11). As a result, 10.9% of microbial variance in vitiligo could be explained by IL-1β [False Discovery Rate (FDR) = 0.005], and 6.4% by VES (FDR = 0.049). Disease duration was responsible for 5.3% of the variance (FDR = 0.093) and associated with an increase in microbial evenness and richness (Shannon and Simpson indexes).

We conducted a correlation analysis between serum metabolome and the gut microbiome and identified seventy-seven strong correlations (*P* < 0.05, Figure 4C). In particular, *Psychrobacter* was markedly linked to 13 differential metabolites, with the highest correlation found with taurine (CC = 0.642, *P* < 0.01) followed by uridine and nicotinamide (positive correlation, *P* < 0.01). Association of *Psychrobacter* with the previously mentioned serum differential metabolite L-NMMA, which was significantly positively correlated with the Shannon index, also reached 0.407 (*P* < 0.05). Moreover, we found that 11 genera, including *Psychrobacter* and *Corynebacterium 1*, were significantly related to linoleic acid *(P* < 0.05), among which *Gemmobacter* exhibited the strongest correlation (LDA = 3.66, CC = 0.733, *P* < 0.01). More importantly, *Psychrobacter*, *Corynebacterium 1*, and *Gemmobacter*, which were closely related to serum metabolites, all had prominent performance in the LEfSe analysis. These findings suggested that various metabolites might be essential molecules for critical species in vitiligo patients’ microbiome.

In order to reveal associations between clinical metadata and microbial community abundance or function, we thus performed MaAsLin analysis of the top genera taxonomy scored by LEfSe (LDA score >2.50 and “importance” >0.021). Results illustrated that smoking had the strongest correlation with *Bilophila* (adjusted *P* < 0.1). Disease duration exhibited notable negative correlations with *Corynebacterium 1* and *Psychrobacter* (adjusted *P* < 0.1). Disease duration also exhibited a positive correlation with *Ruminococcus 2* (adjusted *P* < 0.1). IL-1β, a comparatively sensitive serological marker in vitiligo progression (Bhardwaj et al., 2017), was negatively correlated with *Corynebacterium1*, *Jeotgalibaca*, and *Psychrobacter* (adjusted *P* < 0.1). Diet, sex, and sCD25 showed no remarkable association with different genera (Figure 4D and Supplementary Table 12). Our results showed that *Corynebacterium 1*, *Ruminococcus 2*, *Jeotgalibaca*, and *Psychrobacter* were closely related to the disease status of vitiligo. Other markers such as *Subdoligranulum* and *Escherichia/Shigella* screened by LEfSe were not found to be significantly associated with vitiligo disease status. The results of LEfSe, MaAsLin and machine learning converged into the thesis that *Corynebacterium1* and *Psychrobacter* might be an important prominent marker of vitiligo.

### Combined Predictor Models Based on Taxonomies and Serum Metabolites Reach an Accuracy of More Than 0.9

**FIGURE 5:**
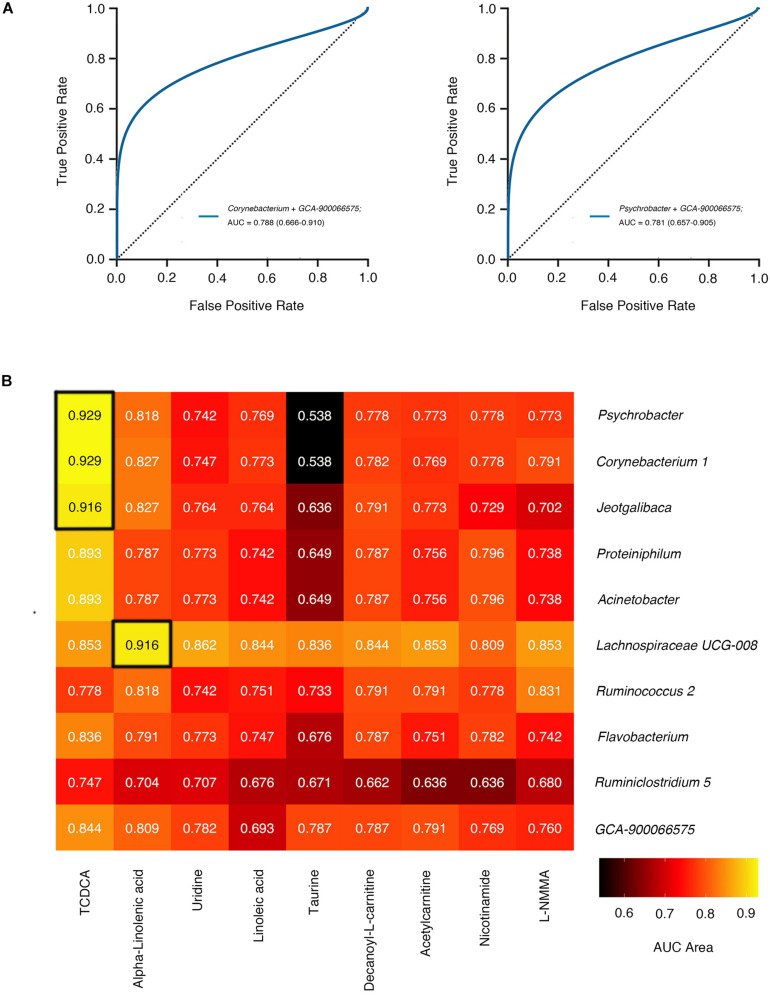
The accuracy of gut microbiome feature joint model. **(A)** The smooth ROC curves model using 10-fold cross-validation for the predictions of microbial genera in our cohort. **(B)** The AUC area of joint prediction models using 10-fold cross-validation combined serum differential metabolites with gut microbiome markers are shown in the heat map. The higher AUC value is illustrated by a lighter color. ROC curves of two genera correspond to the blue line. Abbreviation: AUC, Area Under Curve; ROC, Receiver Operating Characteristic.

We next selected genera from the accumulation of dominant genera screened out by LEfSe (LDA score >2.00) and machine learning classifier (“importance” >0.015) to establish prediction models. The Area Under Curve (AUC) in the Receiver Operating Characteristic (ROC) represents accuracy of models we established. The accuracy of prediction models of mono-taxonomy got to maximum when based on *Parasutterella* (AUC = 0.705, 95% CI 0.57 to 0.84), and models of two taxonomies when based on *Corynebacterium 1* and *GCA-900066575* (AUC = 0.788, 95% CI 0.67 to 0.91, and see in Figure 5A, Supplementary Tables 13, 14).

We went on to explore whether serum metabolites associated with differential microbial structures were better predictors for disease classification. Intriguingly, more than 85% of combined predictor models based on taxonomies and serum metabolites manifested accuracy over 0.7, superior to models based on merely one or two taxonomies. Notably, *Psychrobacter* and TCDCA collaboratively showed a better prediction accuracy (AUC = 0.929), approximately two-fold higher than *Psychrobacter* alone (AUC = 0.466), which indicated a sophisticated interplay between these two factors (Figure 5B). Meanwhile, the prediction model combining *Corynebacterium 1* and TCDCA also achieved high accuracy (AUC = 0.929).

## Materials and Methods

### Study Population and Sample Collection

Advanced non-segmental vitiligo patients (*n* = 30, 12 males and 18 females, mean age 37.2 ± 12.7 years, body mass index/BMI, 21.89 ± 2.96 kg/m^2^) and healthy volunteers (*n* = 30, 12 males and 18 females, mean age 35.2 ± 12.5 years, BMI, 21.62 ± 2.23 kg/m^2^) were recruited in the Department of Dermatology, Xijing Hospital, Fourth Military Medical University from 2018 to 2019. Sex, age, smoking status, and BMI were matched between the two groups to avoid the effects of confounding variables.

All patients developed new lesions in recent 3 months. And all patients developed vitiligo with significant disease activity maintaining for over 3 months and average of disease duration was 6.4 years in our vitiligo patients. Moreover, the VES (van Geel et al., 2018) has been previously validated and it yielded excellent evaluation outcomes. It means the disease becomes more severe with higher white spot area. The results of the VES were 6.2% on average in our study.

Basic information including age, sex and BMI, coupled with a dietary survey was administered and collected from all subjects in the form of questionnaires according to standard procedures. Fecal samples from all participants were freshly collected at hospital/home in MGI Easy Stool Sample Collection kit and frozen at −80°C in multiple aliquots immediately after sampling. For participants, peripheral venous blood samples were drawn in the morning the day after admission and collected as standard venipuncture requested. Blood samples were processed for preparation of serum and plasma samples to apply for downstream metabolomic validations.

Serum IL-1β and sCD25 levels were determined via ELISA. One data point for the result of IL-1β from the control group was missing and we substituted it with the average value. The results showed that serum IL-1β levels were significantly higher in vitiligo group than those in controls, with a *P*-value of 0.001, which were in agreement with previously reported results. However, the sCD25 results showed no significant difference between the two groups (see statistic data in Supplementary Table 1).

#### Assessment of Different Ddietary Habits (Food Intake)

a. Vegetarians (*n* = 23)

Vegetarians were those who led a vegetarian lifestyle (i.e., eat animal food less than once a week), and they were marked as “Veg.”

b. Balanced dieters (*n* = 28)

Balanced dieters mean “average omnivores.” These people who ate what they enjoyed such as “average” Chinese diet had a balanced diet of meat and vegetables.

c. Meat consumers (*n* = 9)

Meat consumers were respondents who lived a meat-eating lifestyle (i.e., always eat animal food, occasionally vegetables).

By comparison, confounding dietary effects were eliminated between patients and volunteers.

### 16S rRNA Gene Sequencing and Data Analysis

Bacterial DNA derived from fecal samples was extracted by means of phenol-chloroform extraction after storage at −80°C. High-throughput sequencing of bacterial 16S ribosomal RNA gene (16S rRNA) amplicons encoding V4 region (150 bp read length, paired-end protocol) was performed using MiSeq Illumina Sequencer. The 16S rRNA sequencing data were analyzed using the Quantitative Insights into Microbial Ecology2 (QIIME2) pipeline (version 2019.4) (Bolyen et al., 2019; Supplementary Figure 5). The proportion of reads at the ASVs or at the level of the genus was used as a measure of relative abundance of each type of bacteria.

Raw sequence data of 16S rRNA gene were demultiplexed and quality filtered using the q2-demux plugin followed by denoising with Deblur (via q2-deblur) (Amir et al., 2017). All ASVs were aligned with mask (Lane, 1991) (via q2-alignment) and were used to construct a phylogeny with FastTree (Price et al., 2010) (via q2-phylogeny). After the collection of samples, α-diversity metrics (Simpson and Shannon indexes), β-diversity metrics [unweighted UniFrac (Lozupone and Knight, 2005)], and Principle Coordinate Analysis (PCoA) were estimated via q2-diversity. Taxonomy was assigned to ASVs using the q2-feature-classifier classify-sklearn naïve Bayes taxonomy classifier against Silva (Quast et al., 2013) 132 99% OTUs from 515F/806R region of sequences.

#### Gneiss (Morton et al., 2017)

Differential abundance analysis was performed by gneiss from 16S data with vitiligo patients. We employed unsupervised clustering via Ward’s hierarchical clustering to obtain Principal Balances. Then we performed the isometric log ratio (ILR) transform which were computed the log ratios between groups at each node in the tree. Next, we conducted a multivariate linear regression on each balance separately and associated microbial abundances with environmental variables. The model is presented as follows:

$$\vec{\text{y}}=\beta \,0\to +\beta \,\text{Group}\to \text{XGroup}\to \beta \,\text{sex}\to \text{Xsex}\to +\beta \,\text{age}\to \text{XAge}\to \beta \,\text{BMI}\to \text{XBMI}\to +\beta \,\text{Smoke}\to \text{XSmoke}\to +\beta \,\text{Diet}\to X\,\mathrm{Diet}\to$$

(Where $\vec{\text{y}}$ represents the matrix of balances to be predicted, βi→ represents a vector of coefficients for covariate i and Xi→ represents the measures for covariate i).

To evaluate the explanatory model of a single covariate, a leave-one-variable-out approach was used. One variable was left out, and then the change in R2 was calculated. A 10-fold cross-validation was performed to avoid overfitting. R2 in the Supplementary Table 8 provided information about how much variance that could be explained by the regression model in the community.

#### Linear Discriminant Analysis Effect Size (LEfSe) (Segata et al., 2011)

To evaluate the differences between groups of 16S data or inferred metagenomes, we used LEfSe analysis in Galaxy interface platform (Afgan et al., 2018). Features significantly discriminating among groups were then subjected to the linear discrimination analysis (LDA) model with a threshold logarithmic LDA score >3.0, which were represented in histogram, as produced by LEfSe with default parameters at the genus level. All LDA values of top 20 microbes shown in our figures were organized in the Supplementary Table 3.

#### Co-occurrence Network Inference

Building co-occurrence networks of the most abundant genera intended to evaluate the microbiota community structure (>0.5% mean relative abundance in the global dataset) using Sparse Correlations for Compositional data (SparCC) (Friedman and Alm, 2012) algorithm. Pseudo *P*-values were calculated through a bootstrap procedure with 999 random permutations and 999 iterations for each SparCC calculation. It was defined as significant association with positive SparCC correlation resulting in a *P*-value < 0.05. The correlation among 177 genera denoted from 614 key ASVs was calculated with a 100-time replication of bootstrap procedure, and subsequently correlation matrices were computed from the resampled data matrices. Closeness centrality values (closeness function) serve to calculate node closeness centrality (node opacity) and Kamada-Kawai layout algorithm was carried out to achieve network visualization.

#### Prediction of Microbiome Functional Profiles

To determine the genomic potential of microbiome with vitiligo patients, we computationally predicted the 16S rRNA gene depending on Tax4Fun (Asshauer et al., 2015) algorithm in R. This software tool takes advantage of obtained data sourced from 16S rRNA gene sequencing to predict the functional profile of a bacterial community based on an existing reference genome database. After normalization for 16S rRNA copy number and total species count, Tax4Fun was used to estimate KEGG Orthologs (KO) abundances.

#### Supervised Machine Learning (Pedregosa et al., 2011; Bokulich et al., 2018)

We applied supervised learning classifiers to predicting the categorical metadata classes of unlabeled samples by learning the composition of labeled training samples. The input samples were randomly split into 2 sets between training and test, which aimed to verify accuracy on a set of samples that was not used for model training. The “Confusion matrix” heat map shown in Figure 3A was the result of one of these random learning and prediction exercises. Our model was trained to predict group classifier for each sample and 10-fold cross-validation was performed during automatic feature selection as well as parameter optimization steps to tune the model. Model accuracy was calculated by comparing each test sample’s predicted value to its true value. The feature selection of high ‘importance’ was shown in Supplementary Table 12.

### LC-MS and Data Analysis

The serum metabolomic profiles of participants were measured using non-targeted LC-MS methods ranging from polar metabolites (e.g., organic acids), lipids (e.g., triglycerides), free fatty acids and bile acids. Metabolites were identified by accuracy mass (<25 ppm) and MS/MS data were matched with our standard database. SIMCA-P 14.1 (Umetrics, Umea, Sweden) was used for Orthogonal Partial Least-squares-discriminant Analysis (OPLS-DA) after Pareto-scaling. And OPLS-DA model was estimated by even-fold cross-validation. Univariate analysis was thereafter performed to validate the significant difference.

Serum samples were slowly thawed at 4°C. Then 100 μL of each sample was took out, 400 μL precooling methyl alcohol/acetonitrile (1:1, v/v) was added and adequately vortexed. The samples were incubated for 60 min at −20°C to precipitate the protein, and then were centrifuged (14000g, 4°C, 20 min). The supernatants were collected and dried under vacuum, and then were stored at -80°C as standbys. Next, they were redissolved in 100 μL acetonitrile/water (1:1, v/v) and adequately were vortexed, and thereafter were centrifuged (14,000 rpm, 4°C, 15 min). The supernatants were collected for LC-MS/MS analysis. Sample separation was performed through an UHPLC (1,290 Infinity LC, Agilent Technologies) HILIC and RPLC. Samples were detected in both ESI positive and negative modes. Analyses were performed using an UHPLC coupled to a quadrupole time-of-flight (AB SCIEX TripleTOF 5600). The raw MS data (wiff. scan files) were converted to MzXML files using ProteoWizard MS Convert and processed using XCMS for feature detection, retention time correction and alignment. Single dimensional statistical analysis includes Student’s t test, Mann-Whitney U test and fold change. According to the OPLS-DA model, VIP value was used to measure the expression pattern of each metabolite on the affecting intensity and explanatory ability. Consequently, different metabolites must reach the requirement of VIP > 1 and P-value < 0.05.

### Statistics Analysis

All statistical analyses were conducted in R (v 3.5.3), SPSS (v 25.0.0.0), Galaxy platform and QIIME2 (Supplementary Figure 6). The model was evaluated through ROC with the calculation of the AUC. The smooth ROC curve was estimated by 10-fold cross-validation and pictured by the pROC R package.

Unordered categorical variables were reported as counts and proportions and analyzed by using the χ2 test or the Fisher exact test, when appropriate. The student’s *t*-test was used for analyzing simple associations in the cohort’s baseline characteristics regarding differences in age and BMI (Age and BMI had Gaussian distributions). And non-normally distributed data were tested using either two-sided unpaired Mann-Whitney *U*-test or Kruskal-Wallis pairwise test.

α-diversity measured by Shannon and Simpson indexes was used to quantify the evenness and richness of gut microbiome. β-diversity that characterizes similarities between samples as a function of microbial composition was used to analyze potential impacts of vitiligo on the balance and recovery of the entire gut microbial ecosystem (Lozupone and Knight, 2005). The correlation between metadata, metabolites and Shannon or Simpson diversity was performed by the Spearman coefficient. The proportion of variance of interpretation in each phenotype on the various microbial composition dissimilarities was measured using Adonis Permutational Multivariate Analysis of Variance (PERMANOVA) (Anderson, 2001; Oksanen et al., 2019) test in QIIME2 pipeline. Multivariate association with linear models (MaAsLin), a multivariate statistical framework, was employed to reveal associations between clinical metadata and microbial community abundance or function (Morgan et al., 2012). The association between microbiome features and disease phenotypes were tested using linear models with Multivariate Association with Linear Models (MaAsLin) in Galaxy platform. In a univariate or multivariate predictive model, samples with any missing values were independently removed in this study, and multivariate logistic regression was used to construct the prediction model. In order to improve the robustness of the model, all samples were predicted by 10-fold cross-validation. The sample population was randomly divided into 10 subsets: 1 for testing and the other 9 for training, and the operation was repeated 10 times.

## Discussion

Our study represents the unique effort to validate vitiligo-associated changes in the interaction between the human gut microbiome and circulating metabolome. Together, our discovery highlighted the implication of the gut-skin axis in vitiligo pathogenesis.

Recently, Ganju et al. (2016) reported that vitiligo skin lesions were in association with a particular distribution of skin microbiome. However, skin microbiome is thought to be highly variable and determined by multi-factors ranging from skin sites and diverse microenvironments. Conversely, the composition of gut microbiome tended to remain stable since the early childhood, though it could change with high specificity in disease state especially toward autoimmune disorders. Additionally, the study of Dellacecca et al. (2020) may provide a clue that ampicillin-induced depigmentation was more related to gut dysbiosis rather than skin dysbiosis.

We tested the biodiversity of gut microbiome in vitiligo and found that higher α-diversity was considered to be vitiligo-specific (Figure 1A), contrasting with some other autoimmune diseases with lower bacterial diversity (Vich Vila et al., 2018; Hidalgo-Cantabrana et al., 2019). The patients with disease duration beyond 5 years demonstrated prominently increased α-diversity (Figure 1B), implicating that the gut microbiome alteration might result from a long-term inflammatory status. Combined with distinct β-diversity, we concluded that the gut microbiome of vitiligo patients was subject to dysbiosis (Figure 1C). The reduced *Bacteroidetes*: *Firmicutes* ratio as a fecal microbial signature in patients with vitiligo compared with healthy controls (Figure 2A) was in line with the situation in IBD (Bamola et al., 2017) and multiple sclerosis (MS) (Cosorich et al., 2017), the latter of which may be attributed to high disease activity and increased intestinal Th17 cell frequency. The *Negativicutes* class, which showed a slow decline as the disease course prolonged, was drastically reduced in ankylosing spondylitis patients (OTU 14148, adjusted *P* = 5.3 × 10–28) (Nayfach et al., 2019), suggesting *Negativicutes* class may play a similar role in a variety of autoimmune diseases. Moreover, *Gammaproteobacteria* class including screened genera *Escherichia/Shigella*, *Psychrobacter*, and *Parasutterella* displayed increased firstly and then decreased (Figure 2B). Interestingly, it was also observed that the enrichment of *Gammaproteobacteria* prompted the microbial alteration in IBD women and even in their infants (Torres et al., 2019), which may suggest the similar potential consequences of abnormal early-life microbiome exposure for patients with vitiligo. Our module of co-occurring genera might indicate the profound imbalances in the inter-species relationship of vitiligo microbial community (Supplementary Figure 3). *Subdoligranulum*, *Parabacteroides*, *Parasutterella*, *Azospirillum Sp. 47*, and *Butyricoccus* were the core nodes of the network, acting as the central hub, indicating that these species had a stronger symbiotic relationship with other species and were more closely related to other species. However, genera such as *Psychrobacter* and *Corynebacterium 1* were at the edge of the coexisting network. We speculated that the metabolic functions of these species might be irrespective of the living conditions that shared with other genera.

Survey of the patients’ gut microbiome might be an essential part for vitiligo non-invasive screening and differential diagnosis. The gut microbiome potentially operative in vitiligo pathogenesis also have notable implications for development of health checkups and health products directed at the dysbiosis. Supervised machine learning enabled the prediction of vitiligo with extremely high accuracy assisted by QIIME2 platform. And characteristic microbes’ outputted by machine learning could be viewed as vitiligo biomarkers. Distinguished gut microbiome of vitiligo patients might be an essential part for vitiligo non-invasive screening and differential diagnosis. The gut microbiome potentially operative in vitiligo pathogenesis also have notable implications for development of health checkups and health products that direct at microbiome. So far, we have obtained several attractive and promising disease-related markers. *Psychrobacter* functions in pathways such as fatty acid biosynthesis, degradation and metabolism (Tribelli and Lopez, 2018), which are consistent with differential metabolites identified in vitiligo patients’ serum metabolome that also involves in fatty acid metabolism pathways (i.e., eicosapentaenoic acid). The characteristics of the vitiligo-associated gut microbiome are shared by extensive autoimmune diseases highly related to potential pathogenic microbes (Lloyd-Price et al., 2019; Torres et al., 2019).

A large number of clinical studies and epidemiological investigations suggest that changes in the environmental status of the host have an essential impact on the gut microbiome (Dominianni et al., 2015; Tamburini et al., 2016; Zmora et al., 2019; Leshem et al., 2020). Previously, internal and external factors, like age, sex, dietary, smoking status, immune state, metabolites and environment, were reported to affect gut microbiome composition in the general population (Dominguez-Bello et al., 2019). Our results support that the clinical variable “Diet (Veg)” contributes the most to the changes in the gut microbiome of the enrolled cohort (Figure 4A). Still, in-depth analysis found that “Diet (Veg)” have no significant effect on the gut dysbiosis in patients with vitiligo (Figures 4B,D). The reason may be that the clinical variable “Diet (Veg)” is not a critical factor in the formation of gut dysbiosis in patients with vitiligo. Cohort studies with larger sample sizes may be needed in the future to confirm this.

Most potential gut microbial markers in vitiligo were significantly associated with differential serum metabolites according to gut microbiome and serum metabolomics association analysis. Currently, it’s widely accepted that metabolites might serve as the preferred carbon source for promoting growth of microbial species, or byproducts of species metabolism released into the blood circulation (Franzosa et al., 2019). Some elevated vitiligo-associated metabolites identified in the present study (Figure 4C) are also reportedly to be risk factors in IBD, including taurine, TCDCA, eicosapentaenoic acid and linoleic acid (Dawiskiba et al., 2014; Lloyd-Price et al., 2019). This indicates that gut microbiome and serum metabolome might operate in similar mechanisms in vitiligo to those in IBD, presumably via igniting immune response.

As a critical component of primary bile acids, TCDCA participates in and affects the bile acid metabolism pathway of microbes (Heinken et al., 2019). At the same time, TCDCA can be biosynthesized into tauroursodeoxycholic acid (TUDCA) (Song et al., 2017), which is an inhibitor of endoplasmic reticulum stress (Keestra-Gounder et al., 2016). Thus, TCDCA might be suppressive in the progression of endoplasmic reticulum stress-induced vitiligo (Park et al., 2019) through bile acid metabolism. The close relationship between microbe and metabolite (Figure 4C) exhibited in the present study might extend the notion of microbiome-host interaction pattern in disease development. Moreover, the integration of gut microbiome and circulating metabolites signals greatly optimized the prediction accuracy of vitiligo in a complementary way (Figures 5A,B). It is predicted that the better microbes and metabolites in the models have the potential to be new biomarkers of disease and may play an important role in the follow-up study of vitiligo mechanism. Nevertheless, microbial markers in vitiligo such as *Psychrobacter*, *Corynebacterium 1*, and *Gemmobacter* are rarely reported in other autoimmune diseases.

Several limitations should be marked when interpreting our results. The deficiency of metagenomics sequencing data limited the analysis of metabolites-relevant functions and assessing the power of microbiome signature. Prospective study with a large (multi-center) cohort and validation study is necessary to verify the proposed biomarkers. Still, the relevancies that we found based on bioinformatics have provided the foundation for the study of cause-effect relationships, which warrants further investigation in mouse models of vitiligo. To disentangle the clear roles of these metabolites by experiment in the year ahead, species should grow in associated metabolites and/or their metabolites are required to be analyzed in a single culture. Of note, the identification of altered gut microbiome composition and functional pathway associated serum metabolites by the present study build a complex etiology network with vitiligo.

## Data Availability Statement

The datasets presented in this study can be found in online repositories. 16S data involved in this manuscript had been deposited in China National Microbiology Data Center (NMDC) with project ID NMDC10010958. All data can be accessed from ftp site ftp://biomirror.im.ac.cn/Gut_microbiome_and_vitiligo/ and http://nmdc.cn/resource/genomics/project/detail/NMDC10010958.

## Ethics Statement

The studies involving human participants were reviewed and approved by the Institutional Ethics Committee, Xijing Hospital, Fourth Military Medical University. The patients/participants provided their written informed consent to participate in this study.

## Author Contributions

CL, SL, and QN: conceptualization and methodology. QN and ZY: software, formal analysis, and data curation. QN, ZY, and YW: validation. QN: investigation and visualization. CL, ZJ, CM, KL, YL, LL, ZH, and TG: resources. QN, YW, JC, and ZY: writing—original draft preparation. QN, YW, ZH, and WZ: writing—review and editing. CL and SL: supervision. CL: project administration and funding acquisition. All authors have read and agreed to the published version of the manuscript.

## Conflict of Interest

The authors declare that the research was conducted in the absence of any commercial or financial relationships that could be construed as a potential conflict of interest.
