# Supplementary material for: Gut Microbial Dysbiosis and Plasma Metabolic Profile in Individuals With Vitiligo
Source: Front Microbiol. 2020 Dec 14;11:592248. doi: 10.3389/fmicb.2020.592248 (PMC7768019; doi:10.3389/fmicb.2020.592248)

Supplementary Figures:

Figure S1. Rarefaction curves of Shannon index. Comparison of average α-diversity (biodiversity) between vitiligo patients (n = 30, depicted in red) and controls (n = 30, depicted in blue) is shown in the average rarefaction curves of Shannon index with increasing sequencing depth.

Figure S2. Pattern of gut microbiome dynamics with VES in vitiligo patients. Mean relative abundances of gut microbiome at the class level dynamically changed with VES, which were tracked using Sankey plots in vitiligo patients. We divided the white patches areas of cases into 4 categories including 0-0.24%, 0.24-2%, 2-10%, and 10-50% according to VES, and observed dynamic shifts in the mean RAs of *Actinobacteria*, *Bacteroidia*, *Clostridia*, *Verrucomicrobiae*, *Erysipelotrichia*. Among them, the mean RAs of *Bacteroidia* and *Verrucomicrobiae* mean RA illustrated a similar trend. Mean RA of *Clostridia* fluctuated, continuously increasing from 7314.8 to 8316.2 and further to 12028.6 and then slightly reducing to 165.8.

Figure S3. Microbial co-occurrence analysis of vitiligo with gut ecosystem. Ecosystem-specific co-occurrence patterns at the genus level were visualized using network diagrams in which microbial taxa (LDA score > 2.20) represent nodes; the presence of a positive co-occurrence relationship based on correlation was represented by an edge for vitiligo patients involved in our cohort. The class to which each genus belongs represents circle color. Each co-occurrence relationship has strong Spearman's correlation coefficient ($P < 0.05$).

Figure S4. Prediction matrix of machine learning classifier at the genus level. 'Confusion matrix' heat map shows the results of classifier accuracy at the genus level. Numbers, which were randomly split by learning methods marked in the figure, represent the size of test set.

Figure S5. 16S sequencing data pipeline on QIIME2. QIIME2, Quantitative Insights into Microbial Ecology2.

Figure S6. Overview of statistical analyses.

Figure S1. Rarefaction curves of Shannon index.

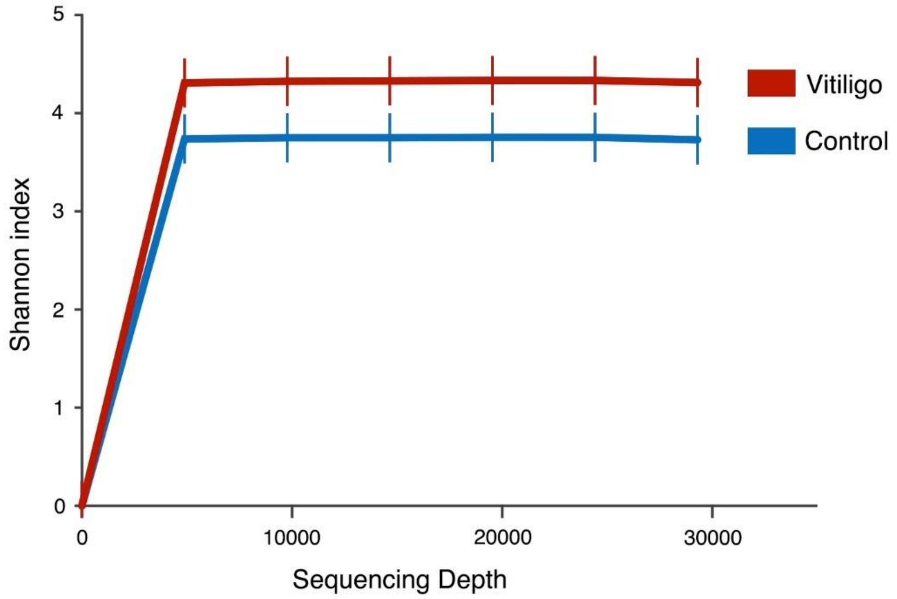

Figure S2. Pattern of gut microbiome dynamics with VES in vitiligo patients.

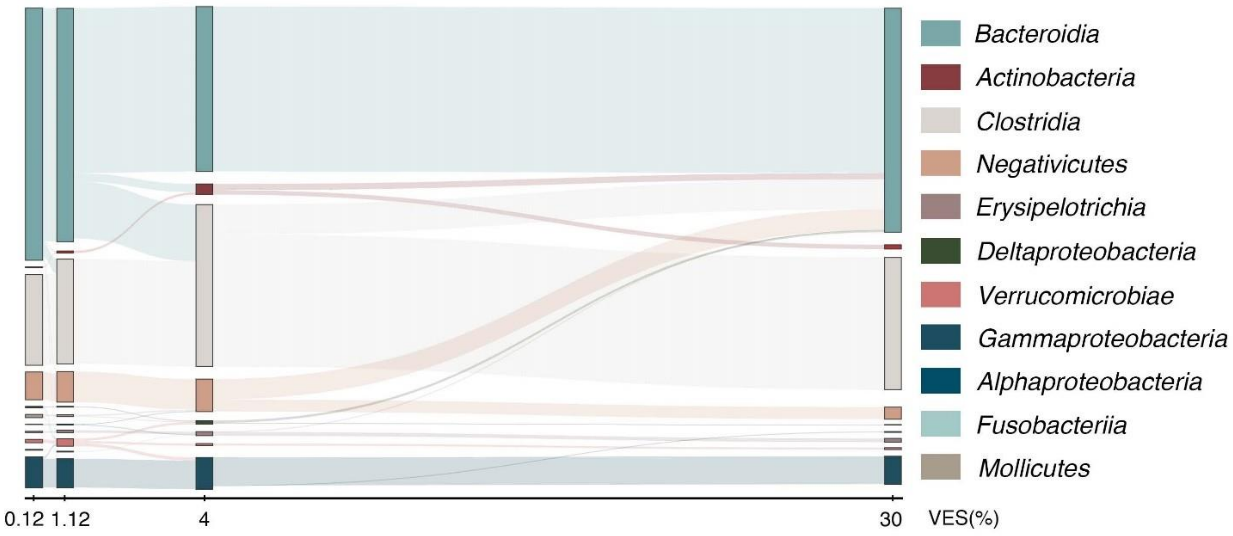

Figure S3. Microbial co-occurrence analysis of vitiligo with gut ecosystem.

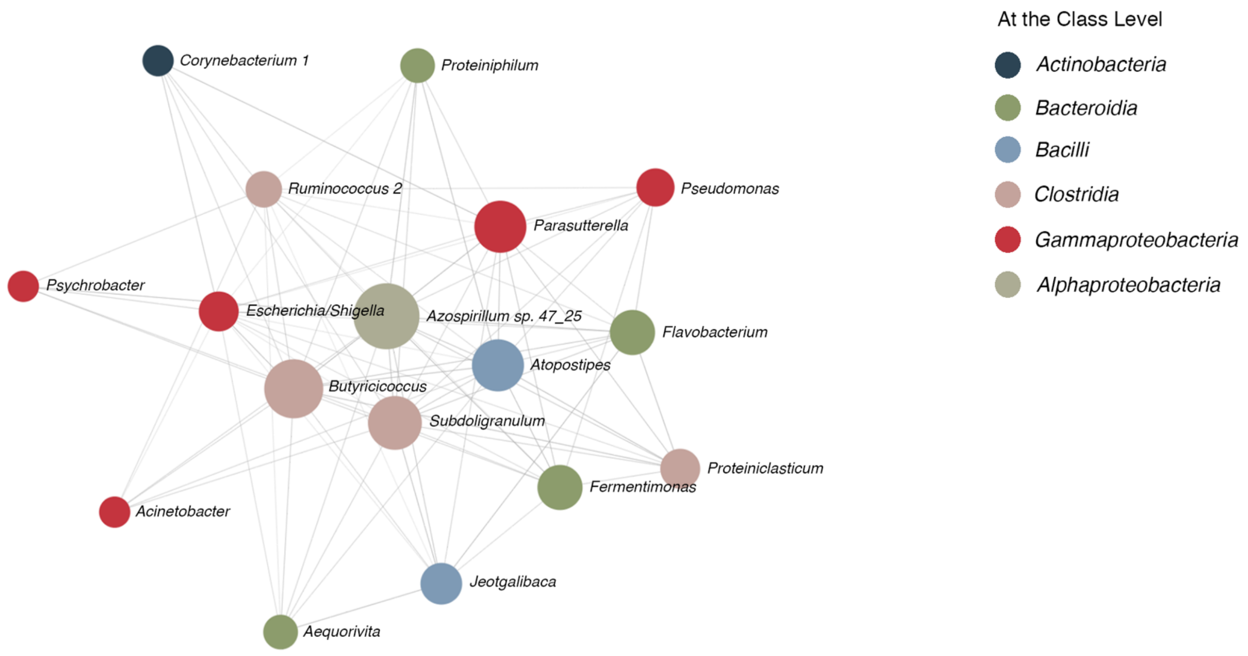

Figure S4. Prediction matrix of machine learning classifier at the genus level.

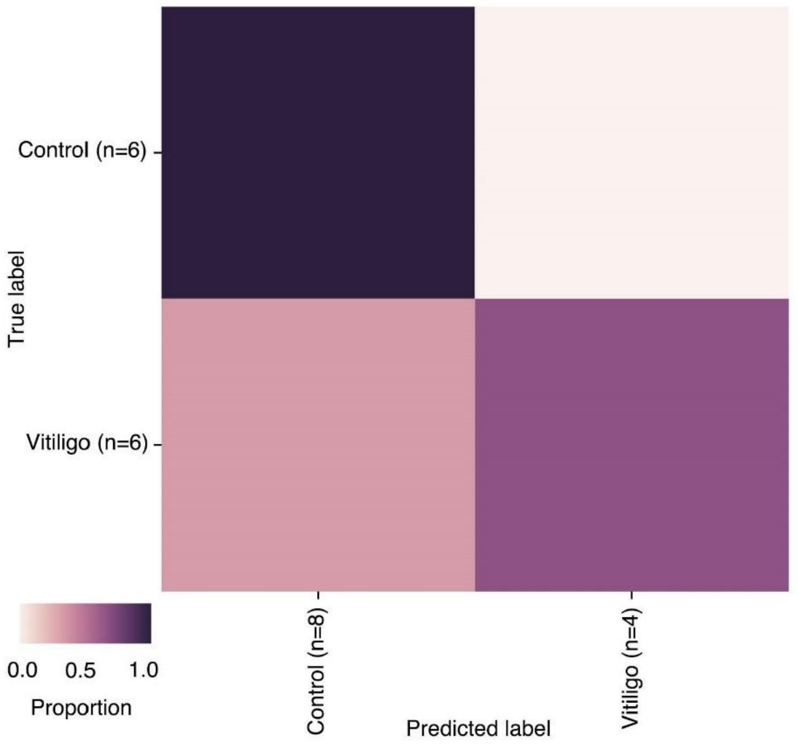

	Control	Vitiligo	Overall Accuracy
Control	1.0	0.0	
Vitiligo	0.333	0.667	
Overall Accuracy			0.833

Figure S5. 16S sequencing data pipeline on QIIME2.

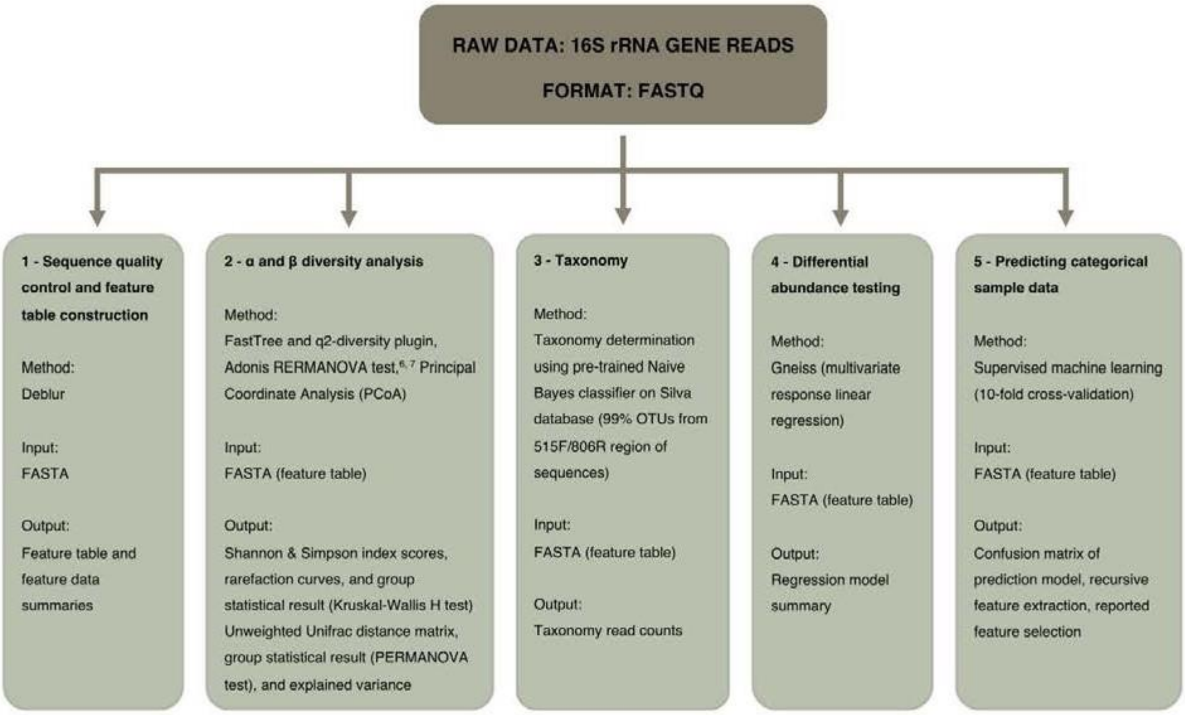

Figure S6. Overview of statistical analyses.

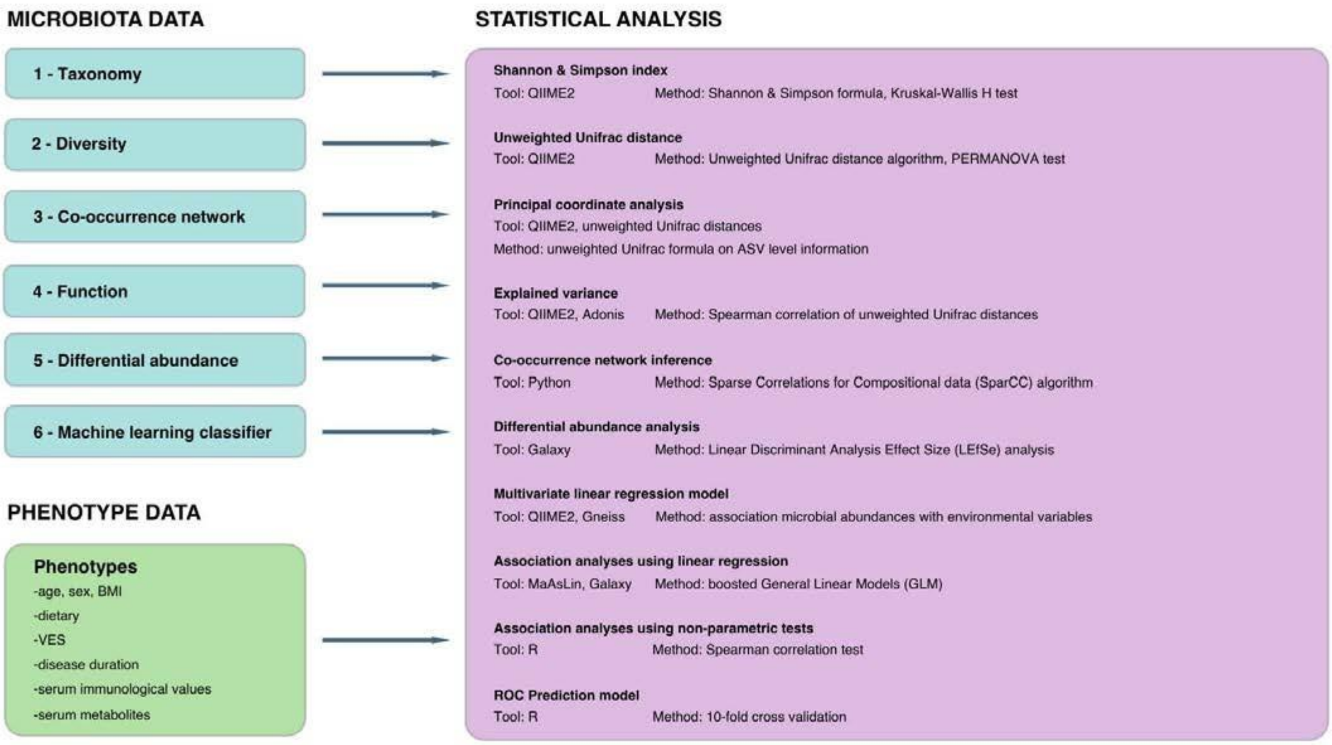

Supplement: Supplementary file 1 [file Data_Sheet_1.pdf]
